# Alignment and Proficiency of Virgin Olive Oil Sensory Panels: The OLEUM Approach

**DOI:** 10.3390/foods9030355

**Published:** 2020-03-19

**Authors:** Sara Barbieri, Karolina Brkić Bubola, Alessandra Bendini, Milena Bučar-Miklavčič, Florence Lacoste, Ummuhan Tibet, Ole Winkelmann, Diego Luis García-González, Tullia Gallina Toschi

**Affiliations:** 1 Alma Mater Studiorum—Università di Bologna, 40127 Bologna, Italy; sara.barbieri@unibo.it (S.B.); tullia.gallinatoschi@unibo.it (T.G.T.); 2 Institute of Agriculture and Tourism, HR-52440 Poreč, Croatia; karolina@iptpo.hr; 3 Science and Research Centre Koper, 6000 Koper, Slovenia; milena.miklavcic@guest.arnes.si; 4 Institut des Corps Gras, 33600 Pessac, France; F.LACOSTE@iterg.com; 5 Ulusal Zeytin ve Zeytinyağı Konseyi, 35100 Izmir, Turkey; ummuhan.tibet@uzzk.org; 6 Eurofins Analytik GmbH, 21029 Hamburg, Germany; OleWinkelmann@eurofins.de; 7 Instituto de la Grasa (CSIC), 41013 Sevilla, Spain; dlgarcia@ig.csic.es

**Keywords:** virgin olive oil, quality, sensory analysis, panel test

## Abstract

A set of 334 commercial virgin olive oil (VOO) samples were evaluated by six sensory panels during the H2020 OLEUM project. Sensory data were elaborated with two main objectives: (i) to classify and characterize samples in order to use them for possible correlations with physical–chemical data and (ii) to monitor and improve the performance of panels. After revision of the IOC guidelines in 2018, this work represents the first published attempt to verify some of the recommended quality control tools to increase harmonization among panels. Specifically, a new “decision tree” scheme was developed, and some IOC quality control procedures were applied. The adoption of these tools allowed for reliable classification of 289 of 334 VOOs; for the remaining 45, misalignments between panels of first (on the category, 21 cases) or second type (on the main perceived defect, 24 cases) occurred. In these cases, a “formative reassessment” was necessary. At the end, 329 of 334 VOOs (98.5%) were classified, thus confirming the effectiveness of this approach to achieve a better proficiency. The panels showed good performance, but the need to adopt new reference materials that are stable and reproducible to improve the panel’s skills and agreement also emerged.

## 1. Introduction

The sensory methodology for virgin olive oils (VOOs) known as the “panel test” was proposed in 1987 [1] and, to date, represents the most valuable approach to assess sensory characteristics and quality for consumer and producer protection [2]. The purpose of the method is to standardize procedures for evaluation of the organoleptic characteristics of VOOs and to establish specific quality grades (extra virgin olive oil—EV, virgin olive oil—V, ordinary virgin olive oil—O, lampante olive oil—L). A group of assessors selected in a controlled manner, suitably trained to identify and measure the intensity of positive and negative sensations, represents the analytic tool of this methodology. A collection of methods and standards has been adopted by the International Olive Council (IOC) for sensory analysis of VOOs. These documents describe the vocabulary that tasters must adopt, the characteristics that the sensory laboratory must possess, the tasting conditions and characteristics of the glass for organoleptic analysis of oils, and the sensory method and rules for the selection, training, and monitoring of skilled virgin olive oil tasters [3,4,5,6,7].

In 1991, the method was included into European regulations and obtained the legal validity for establishing the quality grade of the product that included only three categories of VOOs: EV, V, and L [8]. Since application of the method showed some drawbacks, drawing on its own experience, the IOC made a series of revisions to render the method simpler and more reliable [9].

In 2002, the most important innovation introduced was the application of a statistical index to classify oils according to the median of the main perceived defect (mpd) and the median of the fruity attribute that represents the most important positive descriptor. A limit for the value of the robust variation coefficient, which must be no greater than 20%, was also established. In fact, the use of statistical procedures to analyze sensory data is fundamental, as it provides reliable results that are required for data from other analytical methods [10]. Subsequent amendments and revisions concerned, for example, a list of sensory defects, the specific optional terminology for labeling purposes or tasting conditions, have been adopted up to now [9].

Although it has been responsible for improving the quality of VOOs in the last 28 years, the Panel test is frequently under scrutiny. The problem mainly focuses on the debated classification of borderline oils (EV vs. V, V vs. L), reproducibility of results among different laboratories, the limited number of samples that can be analyzed per day (four samples in each session with a maximum of three sessions per day) and the presence of at least 8–12 trained individuals for each sensory evaluation [6]. Another problem in applying the panel test method depends on the lack of appropriate reference standards for training assessors [11]. In addition, some recent commentaries [12,13] discussed methodological features that should be performed more accurately in order to avoid disagreements between different panels. To overcome this, some strategies were proposed that should be applied during different steps of training of assessors (determination of the group detection threshold, selective trials) or during official tasting sessions (alternative approach to the CVr%) for overcoming these difficulties.

Organoleptic assessment is both a qualitative and quantitative method since its application results in the classification of samples based on the median of the main predominant defect and the presence or absence of the fruity attribute. Consequently, assessors in each panel must be effectively trained for correct classification of samples and for correct recognition of the intensities of perceived attributes.

In this context, the OLEUM project “Advanced Solutions for Assuring Authenticity and Quality of Olive Oil at Global Scale” funded by the European Commission within the Horizon 2020 Programme (2014–2020, grant agreement No. 635690), is engaged in reinforcing the methodology for sensory evaluation through design of a global procedure named the “quantitative panel test”. This approach aims to improve the activity of sensory panels, whose work remains central to ensuring the quality of the product by: (i) reducing the number of samples to be assessed by the sensory panel by establishing chemometric models (calibrated on a large dataset of reliable sensory classified VOOs) that are able to predict assignment of samples to a specific quality grade using rapid instrumental screening methods, which could allow pre-classification with a certain level of probability that can allow the panels to focus more on sensory analysis of uncertain samples; (ii) increasing the panel’s performance by introducing new artificial reference materials validated by a number of sensory panels (six in the case of the OLEUM project) and formulated ad hoc to resemble specific sensory attributes (e.g., rancid and winey); (iii) relating attributes and defects found in VOO with specific molecules (volatile compounds) in order to have an additional qualitative and quantitative tool (quantitation of specific volatile compounds) to support the panel test in confirmatory analyses or in cases of disagreement between panels.

In this regard, some recent works deal with the monitoring of the presence of molecular markers related to specific sensory defects in VOO headspace [14] together with the setting up of chemometric models based on volatile compounds for the prediction of sensory characteristics [15].

The present paper does not aim to illustrate the entire scheme and all the methods involved in the “quantitative panel test”. However, in the framework of the panel test, it highlights possibilities for amelioration and describes the proficiency improvement given by formative training, and the method used to obtain sensory classified samples from analysis of a set of VOOs to be used for calibration of rapid instrumental screening methods. Herein, the results of the sensory evaluation of 334 samples are reported and discussed with the aim to: (i) verify the effectiveness of application of the sensory method to evaluate the quality of the product according to [8] and latter modifications (EV, V, L); (ii) highlight the importance of the strict application of IOC guidelines for quality control methodology (for selecting, training, and monitoring tasters and panels) by the sensory panels in performing organoleptic assessment of VOO; (iii) obtain the most reliable sensory classification of samples in terms of quality grades and intensities of positive (fruity) and negative (main perceived defect/s) attributes; this can be achieved by application of a newly proposed “decision tree” that includes formative reassessments when misalignments on the category or the main perceived defect and/or fruity attribute occur.

Many studies in the literature have discussed the relationship between the official sensory method applied by a trained panel and consumer perception [16,17,18,19,20,21], but to our knowledge there are few studies comparing the results of different trained panels and none aimed to reinforce the application of the official method and increase harmonization among different panels. Specifically, the key elements of this work are: (i) the very large dataset obtained by collecting 334 oils from two olive harvest seasons, representative of the most common olive cultivars, different geographical origins, different sensory profiles, and, especially, the main sensory defects perceived; (ii) the processing of data provided by several panels to obtain a reliable classification by the application of a new decision tree useful for possible correlations with instrumental data and/or for building discriminating models by different instrumental approaches.

## 2. Materials and Methods 

### 2.1. Sensory Panels

Six panels from six different countries were involved in the sensory analysis carried out in the OLEUM project: EUROFINS from Germany, coded as EU; IPTPO from Croatia, coded as IP; ITERG from France, coded as IT; UNIBO from Italy, coded as UN; UP/ZRS from Slovenia, coded as UP/ZRS; and UZZK from Turkey, coded as UZ.

Each panel has some sort of public authority recognition (national authorities; International Olive Council, IOC; national accreditation bodies for EU standards) [22] and takes part in national and international interlaboratory proficiency tests (organized by private or public authorities) and/or IOC interlaboratory comparison. Their sensory activities are focused on evaluation of the grade of quality (quality control), PDO/PGI certification, olive oil competition, and sensory analysis of samples involved in scientific research. The number of samples evaluated each year by the six panels varies from 125 to 1800. The UNIBO panel was responsible for coordinating the activities of panels and for elaboration of sensory data.

### 2.2. VOO Samples

Each sensory panel (EU, IT, IP, UN, UP/ZRS, UZ) was responsible for the sampling (two years: 2016–2017 and 2017–2018 olive harvest seasons) of a possibly balanced number of extra virgin (EV), virgin (V), and lampante (L) samples defined by sensory evaluation, according to EU Regulations [8] and later modifications.

These samples were collected to be representative of the most common olive cultivars, different geographical origins (without restrictions to the national market of each sensory panel), different sensory profiles and, especially, the main sensory defects perceived. Samples were directly requested from olive oil companies under a nondisclosure agreement containing information related to responsibility and confidentiality of data. The selection of the sample set for each year was based on sensory screening: each panel leader, assisted by his/her deputy panel leader, was responsible for applying the official procedure for assessing the organoleptic characteristics of VOOs (according to [8] and later modifications). At the end of the sensory screening, panel leaders sent the results of sample screening to the UNIBO panel leader.

Each olive oil company that agreed to participate in the sampling phase had to provide information related to each commercial sample furnished (date of sampling, geographical origin of olives, olive variety/varieties, PDO, PGI, sanitary state of olives, time and storage conditions of olives before milling, mill location, technology parameters, date of production of the oil, date of start of oil storage, type of storage tank/bottles, oil storage temperature). The need to collect all available information on each sample was the reason why samples were requested from companies, avoiding collection directly on the market. However, the oils collected were representative of possible commercial samples and also so-called borderline samples that can be the object of disagreement between panels in terms of sensory characteristics. The olive oil company indicated a person who was responsible for oil sampling among its employees, who followed and applied guidelines for oil sampling [23,24].

For each sample, a volume of 7 L was requested from the olive oil company and collected inside adequate tins or bottles. In case of a batch produced and packed (oil already bottled), the responsible person selected by the olive oil company had to collect the volume required (7 L) taken with a random selection of bottles. The panel leader assisted by his/her deputy panel leader was responsible for managing the laboratory samples. The olive oil company dispatched the packaged samples to the panel leader, who organized the preparation of laboratory samples (after proper homogenization, using a 0.5 L tin), their label codes, and shipment to all the OLEUM partners involved (carried out in the shortest time possible by tracked shipments). Sample codes summarized the basic information: partner acronym (responsible for the sampling) and number (progressive for two years, related in unique way for each sample).

The samples collected for each year (first year, 180 oils; second year, 154 oils) were divided into four subgroups and their sensory evaluation as well as sensory results were planned over time by the UNIBO panel. All samples, stored in the lab at 10–12 °C, were reconditioned at room temperature for 6–8 h before preparing samples for sensory analysis.

### 2.3. Sensory Analysis

The panel test method was carried out using six OLEUM panels. Positive and negative descriptors were evaluated according to the official procedure ([8] and later modifications). The intensity of each attribute was graded by assessors using a continuous unstructured line scale of 10 cm. Each 15 mL sample was tasted at 28 ± 2 °C in a tasting booth, regulated in terms of shape and equipment [4]. Each panel leader collected the profile sheets completed by each taster (8–12) from his/her panel, reviewed the intensities assigned to the different attributes, and inserted the sensory data in the IOC Excel program for statistical elaboration based on the calculation of the median. The robust coefficients of variation (CVr%) were calculated and validated ([8] and later modifications).

Moreover, with the aim to monitor and possibly improve the performance of panels, after elaboration of each subgroup of sample data, the UNIBO panel, being responsible for the sensory activities, adopted and applied the quality control procedures to check the validity of the results obtained by OLEUM panels in agreement with IOC guidelines for the quality control of virgin olive oil panels revised in 2018 [25], specifically: (i) z-score estimation was conducted for each sensory panel (IOC z-score and OLEUM z-score) to estimate the panel’s trueness; (ii) available IOC standards and other materials characterized (samples from previous IOC proficiency tests) were provided to each panel for training purposes; (iii) replicate analysis of three samples selected between the entire set of samples, to estimate panel precision, was performed.

### 2.4. Statistical Analysis

For processing sensory data from the assessors of each panel (by each respective panel leader), the IOC excel spreadsheet was applied according to official methodology ([6,8] and later modifications). Sensory data from each panel were processed (by the UNIBO panel leader), and after application of the proposed decision tree, the coefficient of variation (CV), was calculated [26] (dataset). A limit for the CV based on its frequency distribution was also proposed to check the level of variability. The CV frequency distribution was also expressed as cumulative probability by the *t*-test (Student’s test distribution).

For control of the performance of the panel, estimation of both precision and trueness of panels was performed according to IOC guidelines [25]. The estimation of the precision of panels was made during the procedure of replicate analysis by the calculation of both normalized error (En) and repeatability number (rN), whereas control of panel trueness was obtained by z-score estimation.

## 3. Results and Discussion

### 3.1. The Decision Tree

For the “quantitative panel test”, it was very important to classify samples to reach agreement (among the six panels involved) on sensory characteristics (in terms of intensity of positive/negative attributes), thus providing useful information for instrumental analysis.

**Figure 1:**
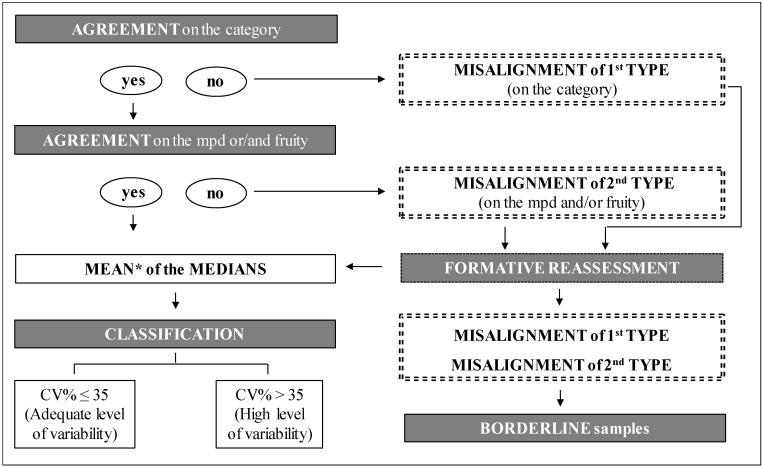
Decision tree adopted for statistical processing of sensory results provided by the six panels. mpd = main perceived defect. * Mean value calculated on the median values obtained by OLEUM panels for mpd and the fruity attribute.

For this specific objective, the classification of samples based on the evaluation data provided by the six panels was elaborated by applying a decision tree (Figure 1), a new tool for categorization of VOOs.

The adopted decision tree is based on the agreement (more than 50% of panels) on the category and on the median of the intensity of the main perceived defect (mpd) and/or of the fruity attribute. If one of these two agreements was not reached, a first or second type of misalignment occurred, and the sample was not classified. Following the flow of the decision tree, the UNIBO panel leader first checked whether the sensory data provided by at least four out of six panels defined the sample as belonging to the same quality grade; if yes, agreement on the mpd was also checked, while in the negative case, formative reassessment was required.

**Figure 2:**
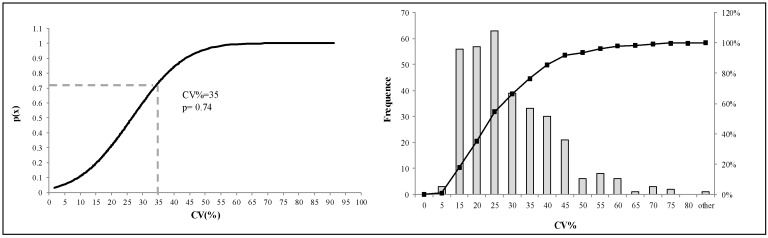
Control of the level of variability of values obtained by application of the decision tree based on the frequency distribution of CV%. CV% = variability of the median values with respect to the mean value. The frequency distribution was also expressed as cumulative probability by *t*-test (Student’s test distribution).

If the desired agreement was met for both criteria, it was possible to proceed with calculation of the mean of the medians (provided by each panel) for classifying the samples. The coefficient of variation (CV%) was applied and considered satisfactory if ≤35% (adequate level of variability). The adoption of 35% as upper limit of CV was selected by observing the frequency distribution of all CV% values registered for the mpd and fruity attribute for the set of samples analyzed. The frequency distribution was also expressed as cumulative probability (*p* = 0.74) applying the *t*-test (Figure 2).

Cerretani and co-workers investigated the relationship between sensory and chemical composition of VOOs to assess correlations between sensory attributes and minor components [27]; in this study, sensory attributes were assessed by four panels (two Italian and two Spanish) employing a total of 59 tasters, and the median values for each VOO evaluated by panels were used as the final input for statistical analysis. In our work, the mean of the medians provided by each panel was considered. The median represents the midpoint of an ordered set of odd numbers or the mean of two midpoints of an ordered set of even numbers. It is, therefore, a robust tool since it is not influenced by outliers; considering that it was already applied by each panel individually, the mean of the medians was also considered more appropriate for comparison of results of panels and for monitoring performance.

The decision tree was applied to the entire set of 334 oils and, in case of misalignments, samples were reassessed in a sensory session (formative reassessment) where each panel was provided with the available IOC reference materials and certified oils evaluated by at least three accredited panels (sent by the UNIBO panel) to improve the identification of any defects and assessment of their intensity. The reassessments were done in a blind way (no information related to the type of misalignments were provided to panel leaders), again applying the organoleptic assessment method, but without open discussion of the attributes between assessors.

During the first year of the project, 176 of 180 oils were classified, and only four misalignments occurred (Table S1a–d); in summary, 152 of 180 samples were immediately classified, and 28 samples were reassessed since first- and/or second-type misalignments occurred (14 samples for each type of misalignment). At the end of formative reassessment, 176 samples were classified (54 EV, 76 V, and 48 L), but classification was not possible for four samples (UN_10, UP_14, EU_29, and UN_32) since agreement among four of six panels was not reached. Specifically, disagreement on the category (V/L) was obtained for UN_10 and UP_14, but for both, fusty-muddy sediment and rancid were perceived by at least four of six panels, indicating these samples as representative of borderline samples; on the other hand, for samples EU_29 and UN_32, an agreement on the category (V) was reached, but not on the identity of mpd due to the presence of more than one defect (fusty-muddy sediment, musty, winey, frostbitten olives, rancid were indicated for EU_29; fusty-muddy sediment, frostbitten olives, rancid were indicated for UN_32), but none were perceived by at least 50% of the panels.

The sensory evaluation of oils from the second sampling (2017/2018 oil campaign), as well as the application of the decision tree, allowed the classification of this set (154 oils) as follows: 69 classified as EV, 51 classified as V, 33 classified as L; one sample was not classified due to an anomalous lemon smell (ZRS_1) and was therefore excluded from the set (Table S2a–d). For 17/154 oils, misalignments of first or second type were achieved (15 and 2, respectively) but, after formative reassessment, all samples were classified by OLEUM panels.

A recent comparative study [28] on a panel test made by nine IOC recognized panels (five from Italy, two from Spain, one from Greece, and one from Slovenia) and chemical analysis of commercial olive oils (16 samples) reported that the sensory methodology works well in case of extremely good olive oils, but not for common commercial ones, and therefore it should be applied only for Protected Designation of Origin (PDO) and other peculiar EVs. Results from the present work, carried out on a large set of commercial VOOs, are in disagreement with those of Circi et al. [28]. The panel test is an official method that has been used to assess improvement in the quality of VOOs since 1991 up to now and provides information on sensory characteristics (intensities of fruity, bitter, and pungent; presence of more than one defect) that are difficult to obtain using a single instrumental approach. The strict application of IOC guidelines for training and quality control of panels and some improvements in the training of a sensory panel, such as the availability of new reference materials that are stable and reproducible, is crucial to increase the reliability of a method to apply a group of assessors as an analytic tool.

### 3.2. The Panel’s Performance

The UNIBO panel, responsible for statistical elaboration of the sensory results, in agreement with the guidelines of IOC document T.28 revised in 2018 [25], summarized the z-score (satisfactory, questionable or unsatisfactory results) for each subgroup of samples from each year and sent it to panel leaders to help them in monitoring the performance of their own panel and to adopt any corrective actions.

The same method adopted by the IOC during its proficiency test (IOC z-score) was applied; it was calculated using: (i) the median (Me) of the predominant defect (the intensity of predominant defect was considered regardless the type of defect that could be different between the six panels) and/or the fruity attribute detected by each panel; (ii) the great median (assigned value, GM) calculated as median of the medians for the predominant defect or for the fruity attribute (detected by all panels as consensus value); (iii) the standard deviation (ơ obj) of the scores calculated from IOC historical data (±0.7). A slightly modified version of this method (OLEUM z-score) was also adopted; the only difference from the previous one was, in case of V and L categories, the use of the median (Me) of the defect identified as predominant by consensus of the panels (even if it was not the predominant defect for each panel).

**Figure 3:**
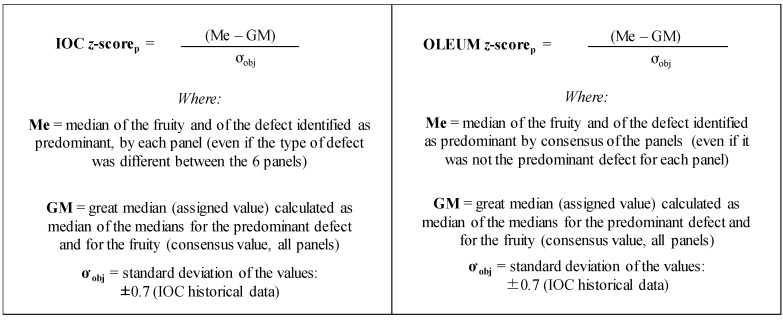
Formulas of the two methods used to calculate the z-score (IOC and OLEUM).

Therefore, the intensity, and also the type of the mpd, was considered in the OLEUM version of the z-score to obtain a reliable dataset for comparison with instrumental data (e.g., in OLEUM for developing screening methods based on the analysis of volatile compounds). The detailed formulas of both the methods used to calculate the z-score are shown in Figure 3.

**Figure 4:**
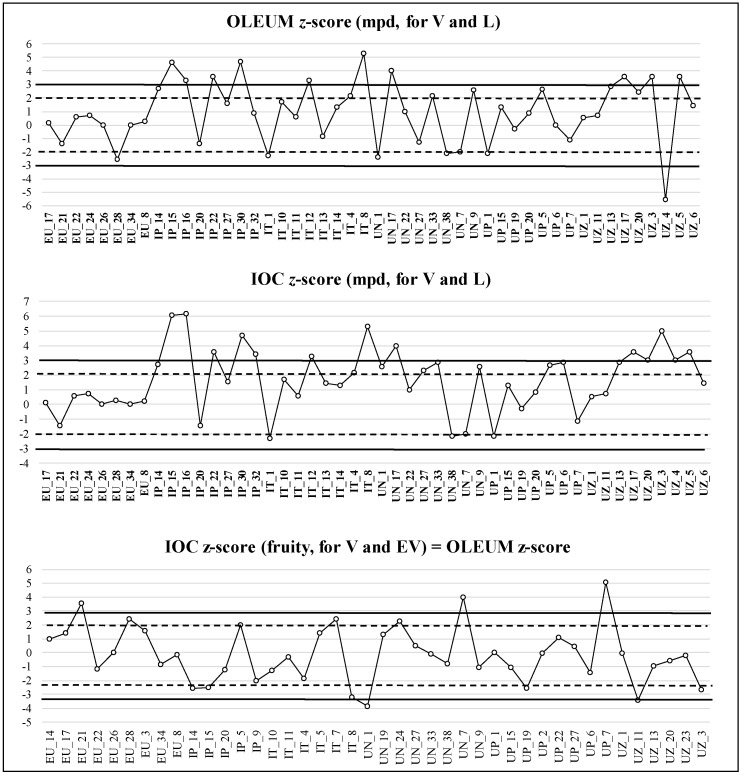
Example of z-score graph for estimation of panel performance, calculated on 60 samples from the subgroup of the first sampling year (180 samples). Criteria of acceptance: |z| ≤ 2, performance was satisfactory; 2 < |z| ≤ 3, performance was questionable; |z| > 3, performance was considered unsatisfactory. The z-scores were calculated for median of the main perceived defect (for V and L category) and for the median of fruity attribute (for V and EV category).

**Figure 5:**
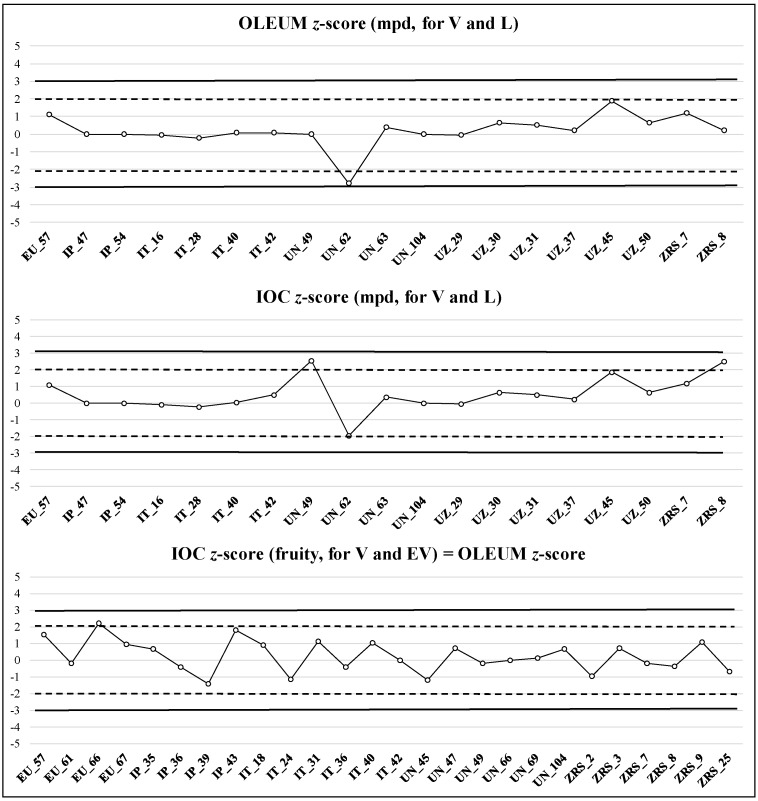
Example of z-score graph for estimation of panel performance, calculated on 38 samples from the third subgroup of the second sampling year (154 samples). Criteria of acceptance: |z| ≤ 2, performance was satisfactory; 2 < |z| ≤ 3, performance was questionable; |z| > 3, performance was considered unsatisfactory. The z-scores were calculated for median of the main perceived defect (for V and L category) and median of fruity attribute (for V and EV category).

Results of the z-score estimation were illustrated by quality control charts, as part of internal quality control. Some examples of panel performance evaluation are reported in Figure 4 and Figure 5; the vertical axis represents the z-score and the horizontal one identifies the sample codes.

The z-score has positive or negative values and was calculated for both fruity (for EV and V category) and negative sensory attributes (for V and L category); the central value is zero, the warning limits for the index are ±2, and the action limits are ±3. The interpretation is the same for both the methods applied (IOC and OLEUM): if |z| ≤ 2, performance was satisfactory; if 2 < |z| ≤ 3, performance was questionable; finally, if |z| > 3, performance was considered unsatisfactory. Each panel leader, observing this chart, had to define any corrective or/and preventive actions taken if a result is outside of the limits or if several consecutive results are obtained at the same side (positive or negative) of the central value (bias) [25]. The results obtained verified that the approach using the z-score represents a very useful tool to evaluate the trueness of the panel over time.

An example of panel performance reported in Figure 4 showed that, in the case of OLEUM z-score for the mpd (V and L), the panel obtained 25 of 48 satisfactory results, 12 questionable, and 11 unsatisfactory, whereas in the case of IOC z-score, 23 of 48 satisfactory, 14 questionable and 11 unsatisfactory results were obtained. In the case of IOC z-score for fruity attribute (V and EV), the panel obtained 29 of 42 satisfactory results, 7 questionable, and 6 unsatisfactory. These results highlight a trend of the panel to more frequently use higher values of the scale for the intensity of mpd or fruity attribute than the GM value (median of the medians of six panels); moreover, in some cases, the presence of a z-score lower than -2 indicated the lack of intensity recognition of the mpd or of the fruity attribute. The second example (Figure 5) showed that for the mpd (V and L), the panel obtained 18 of 19 satisfactory results and 1 questionable result for OLEUM z-score, while obtaining 17 of 19 satisfactory results and 2 questionable results for IOC z-score.

In the case of IOC z-score for the fruity attribute (V and EV), the panel obtained 25 of 26 satisfactory results and 1 questionable result. Overall, the panel showed good performance, although the verification of samples in which the z-score is questionable, using both the panel results and those provided by all panels (by the application of the decisional tree), was suggested in the feedback sent to the panel leader. The estimation of z-score was consistent in evaluating the performance of sensory laboratories over time. Its application in this study showed a progressive, greater convergence of results passing from the first to the second sampling and allowed identification of the critical aspects of the performance of each panel and definition of suitable actions for improvement.

In addition to the z-score estimation, during the second year of sampling, the control of the panel’s precision was also performed by using replicate analysis. The repeatability of panels was controlled by comparing the medians obtained on three samples in duplicate and determining whether the results are homogenous and, therefore, statistically acceptable.

Specifically, three pairs of identical samples were sent to the panels with different codes (blind conditions) (UN_44 = UN_55, UN_59 = UN_60, and UN_66 = UN_69) and the level of agreement between intensity values expressed for the same sample during independent evaluations was estimated by calculating the repeatability number (rN) and normalized error (En), whose acceptability limits are ≤2 and ≤1, respectively [25] (Table 1).

In general, the panels showed good repeatability. In the case of the first pair of samples (UN_44 = UN_55, category V), in fact, the values of both parameters (En and rN) were below the suggested limit for good performance; for the second pair of samples (UN_59 = UN_60, category V), the least satisfactory performances were achieved: three panels showed values above these limits (2, 3 and 6), highlighting the need for additional training to improve performance. Finally, for the third replicated sample (UN_66 = UN_69, category EV), only one panel registered values above the limits due to different intensity of the fruity attribute in the two sessions and therefore was not considered repeatable. These indices are based on the evaluation of the correct intensity of the mpd or fruity attribute (and therefore the product quality grade) by each panel and do not take into account the type of defect; results from the application of the decisional tree were consistent for the correct classification of samples, but not for the mpd (UN_44 fusty-muddy sediment, UN_55 rancid, UN_59 brine, UN_60 winey). The inconsistency in the nature of mpd was probably due to more than one defect present in the sample and with similar intensities; in addition, brine and winey usually go together.

## 4. Conclusions

This work aimed to reinforce the methodology for sensory analysis of VOOs through adoption of supporting tools for training and monitoring of sensory panels. The results obtained from the sensory evaluation carried out by the six panels involved in the OLEUM project on a set of 334 samples confirmed the effectiveness of the application of the panel test. However, at the same time, it also confirmed that there are some critical issues related to questionable results in the case of: (i) borderline oils (between two product categories); or (ii) misalignments on the main perceived defect by panels when more than one negative attribute was present in the oil. The adoption of a decision tree based on the agreement of a category, main perceived defect, and application of formative reassessment in case of misalignments using the same reference materials (samples already classified by the six panels with an high agreement) allowed for reliable classification of oils that, at first evaluation, were borderline. Only 45 of 334 oils were reassessed (formative reassessment) and 41 of 45 samples were definitively classified, confirming the importance of alignment between panels, which can be achieved by sharing the same sensory reference materials. In fact, sensory information on both quality grades of samples and main perceived defect/s is fundamental for testing possible correlations with physical–chemical data and/or for building classification models; in this way, instrumental screening approaches can allow for a reduction in the number of samples that have to be assessed by panels, excluding, for example, oils defintely classified by chemometric models as extra virgin or lampante, focusing the sensory analysis on samples that are not classified or classified with a low probability. This thus reduces the number of samples to be assessed by the sensory panel. The data provided by the panels were also used to verify performance in terms of discriminating capacity, agreement between panels, and accuracy of results by applying some of the procedures reported in the IOC guide for internal quality control of sensory laboratories. In general, the panels showed very good (sometimes excellent) performance even if, in some cases, problems were noted that were related to the use of the scales, lack of recognition of some sensory defects, or intensity values that were too distant between panels for the same sample, especially in the case of oils in which more than one defect was perceived. The large set of samples evaluated over 2 years allowed estimation of the performance of the panel test: the utility and peculiarity of this official method is undisputed, also considering that it has definitively improved the quality of VOOs over the last 28 years, opening the possibility to have a wide range of excellent oils with a deserved added value on the market. On the other hand, to improve its effectiveness, it is necessary that the sensory panels perform organoleptic evaluation by applying specific guidelines [6] and quality control of panel performance [25] in a rigorous manner. To enhance panel skills in recognizing, identifying, and quantifying sensory attributes, the use of new reliable reference materials is of absolute necessity. They could be both “synthetic”, resembling a single negative attribute (e.g., rancid or viney-winegary ) or biotechnologically formulated, in the latter case being closer to actual virgin olive oils. The first type could be used to overcome some of the limitations of the natural matrix and offer advantages such as feasible preparation in each laboratory (open access composition), reproducibility over time, possibility of purchase, and therefore diffusion and availability for the global market. Even the cultural aspects related with knowledge of the sensory aspects of VOOs, i.e., the global recognition of its positive/negative attributes, could also be facilitated by the availability of these “simplified” materials; the formulation and validation of two of these “synthetic” sensory reference materials (rancid and winey-vinegary ) are still in progress within the framework of the OLEUM project. On the other hand, the use of the OLEUM decision tree could be an adequate instrument to classify natural sensory reference materials, for example, those obtained by biotechnological processes (programed fermentations for fermentative defects) or oxidation (for the nonfermentative rancid defect), the availability of which is also fundamental to achieve alignment between panels, thus reducing cases of discordant classifications, which is of vital importance for global trade and product reputation.

## Figures and Tables

**Table 1:** Values of repeatability number (rN), normalized error (En) of each panel for the predominant defect (d) or fruity attribute (f) and suggested limits for these parameters, calculated on the three pairs of samples (UN_44/UN_55, UN_59/UN_60, UN_66/UN_69) evaluated in the replicate analysis (blind conditions).

Panels	UN_44 = UN_55		UN_59 = UN_60		UN_66 = UN_69	
	**En_d_**	**rN_d_**	**En_d_**	**rN_d_**	**En_f_**	**rN_f_**
**1**	0.3	0.4	0.3	1.3	2.0	14.4
**2**	0.3	0.4	1.2	5.3	0.6	1.4
**3**	0.2	0.1	1.2	5.1	0.7	2.0
**4**	0.1	0.1	0.5	1.1	0.6	1.2
**5**	0.3	0.3	0.4	0.6	0.7	2.0
**6**	0.1	0.1	1.2	5.8	0.1	0.0
**Limits**	**≤1**	**≤2**	**≤1**	**≤2**	**≤1**	**≤2**

## Supplementary Materials

The following are available online at https://www.mdpi.com/2304-8158/9/3/355/s1, Table S1 (a–d): Sensory results of samples from the first year, Table S2 (a–d): Sensory results of samples from the second year.
