# Supplementary material for: Alignment and Proficiency of Virgin Olive Oil Sensory Panels: The OLEUM Approach
Source: Foods. 2020 Mar 19;9(3):355. doi: 10.3390/foods9030355 (PMC7143338; doi:10.3390/foods9030355)
Supplement: Supplementary file 1 [file foods-09-00355-s001.pdf]

Table S1a. Sensory results (expressed as mean of the medians of intensities provided by each panel) of samples from the first year (180 oils subdivided in 4 sub-groups). *n.c*, not classified; Mpd, main perceived defect. First sub-group.

Sample code	Selection n.	Formative reassessment	Quality grades	Mpd (intensity and type)	Fruity (intensity)
EU_3	1	no	EV	0.0	4.9
EU_8	1	no	V	1.8 (fusty-muddy sediment)	2.0
EU_14	1	no	EV	0.0	3.8
EU_17	1	no	V	1.6 (fusty-muddy sediment)	2.9
EU_21	1	no	V	1.9 (fusty-muddy sediment)	2.1
EU_22	1	*yes*	V	2.2 (fusty-muddy sediment)	2.7
EU_24	1	no	L	7.6 (fusty-muddy sediment)	0.0
EU_26	1	no	V	2.3 (brine)	1.9
EU_28	1	no	V	1.9 (winey-vinegary)	2.6
EU_34	1	*yes*	V	2.1 (fusty-muddy sediment)	1.8
IP_5	1	no	EV	0.0	4.8
IP_9	1	no	EV	0.0	3.1
IP_14	1	*yes*	V	1.7 (musty)	2.1
IP_15	1	no	V	1.8 (fusty-muddy sediment)	1.9
IP_16	1	no	L	4.5 (fusty-muddy sediment)	0.0
IP_20	1	*yes*	V	1.9 (rancid)	2.9
IP_22	1	*yes*	L	4.7 (rancid)	0.0
IP_27	1	no	L	8 (rancid)	0.0
IP_30	1	no	L	4.5 (rancid)	0.0
IP_32	1	no	L	4.3 (fusty-muddy sediment)	0.0
IT_1	1	no	L	3.8 (winey-vinegary)	2.1
IT_4	1	*yes*	V	2.3 (fusty-muddy sediment)	2.2
IT_5	1	no	EV	0.0	5.4
IT_7	1	no	EV	0.0	4.7
IT_8	1	no	V	1.7 (fusty-muddy sediment)	2.4
IT_10	1	no	V	1.8 (fusty-muddy sediment)	2.9
IT_11	1	no	V	2.1 (fusty-muddy sediment)	2.8
IT_12	1	no	L	4.4 (fusty-muddy sediment)	1.6
IT_13	1	no	L	4.6 (fusty-muddy sediment)	0.0
IT_14	1	no	L	4.8 (fusty-muddy sediment)	0.0
UN_1	1	no	V	2.4 (frostbitten)	2.3
UN_7	1	no	V	1.7 (winey-vinegary)	3.1
UN_9	1	no	V	2.5 (fusty-muddy sediment)	1.9
UN_17	1	no	L	6.6 (musty)	0.0
UN_19	1	no	EV	0.0	5.2
UN_22	1	no	L	5.5 (fusty-muddy sediment)	0.0
UN_24	1	no	EV	0.0	3.8
UN_27	1	no	V	1.2 (fusty-muddy sediment); 1.2 (musty); 1.2 (rancid)	2.2
UN_33	1	no	V	1.4 (fusty-muddy sediment)	2.2
UN_38	1	no	V	2.1 (brine)	3.3
UP_1	1	*yes*	V	1.6 (rancid)	2.8
UP_2	1	no	EV	0.0	4.0
UP_5	1	no	L	4.5 (rancid)	0.0
UP_6	1	no	V	2.3 (winey-vinegary)	3.4
UP_7	1	no	V	2.0 (fusty-muddy sediment)	3.0

UP_15	1	no	V	2.2 (rancid)	2.8
UP_19	1	***yes***	V	2.3 (fusty-muddy sediment)	2.1
UP_20	1	no	L	5.5 (rancid)	0.0
UP_22	1	no	EV	0.0	3.5
UP_27	1	no	EV	0.0	5.2
UZ_1	1	no	V	1.6 (fusty-muddy sediment)	2.7
UZ_3	1	no	V	2.0 (musty)	1.9
UZ_4	1	no	L	4.1 (musty)	1.4
UZ_5	1	***yes***	L	4.9 (rancid)	0.0
UZ_6	1	no	L	7.3 (fusty-muddy sediment)	0.0
UZ_11	1	no	V	2.6 (fusty-muddy sediment)	2.8
UZ_13	1	no	V	1.8 (rancid)	2.2
UZ_17	1	no	L	6.5 (fusty-muddy sediment)	0.0
UZ_20	1	***yes***	V	1.7 (rancid)	1.6
UZ_23	1	no	EV	0.0	3.5

Table S1b. Sensory results (expressed as mean of the medians of intensities provided by each panel) of samples from the first year (180 oils subdivided in 4 sub-groups). *n.c*, not classified; Mpd, main perceived defect. Second sub-group.

Sample code	Selection n.	Formative reassessment	Quality grades	Mpd (intensity and type)	Fruity (intensity)
EU_1	2	no	EV	0.0	4
EU_7	2	no	L	5.1 (musty)	0
EU_23	2	no	V	2.7 (fusty-muddy sediment)	2.2
EU_31	2	no	V	1.2 (fusty-muddy sediment)	2.5
EU_33	2	no	V	2.2 (fusty-muddy sediment)	2.3
EU_36	2	no	V	1.7 (rancid)	2.4
IP_1	2	no	EV	0.0	4.5
IP_8	2	no	EV	0.0	3.5
IP_12	2	no	L	5.4 (fusty-muddy sediment)	0
IP_13	2	no	L	4.4 (fusty-muddy sediment)	0
IP_24	2	no	L	5.2 (fusty-muddy sediment)	0
IP_25	2	no	L	4.5 (fusty-muddy sediment)	0
IT_2	2	no	EV	0.0	4.2
IT_9	2	no	EV	0.0	3.6
UN_2	2	no	EV	0.0	5
UN_3	2	no	EV	0.0	4
UN_15	2	no	L	4.2 (musty)	0
UN_20	2	no	V	1.4 (winey-vinegary)	2.6
UN_28	2	no	EV	0.0	4.5
UN_32	2	***yes***	V	***Misalignment on mpd***	/
UN_37	2	no	L	4.1 (brine)	1.5
UN_40	2	no	V	2.6 (fusty-muddy sediment)	2.6
UN_41	2	no	EV	0.0	3.2
UP_3	2	no	EV	0.0	4.8
UP_10	2	no	L	4.5 (rancid)	0
UP_11	2	no	EV	0.0	4.4
UP_12	2	no	V	2.1 (fusty-muddy sediment)	2.4
UP_23	2	no	V	2 (fusty-muddy sediment)	2.4
UP_28	2	no	V	1.9 (musty)	2.5
UP_30	2	no	V	2.3 (rancid)	2.1
UZ_2	2	no	EV	0.0	2.5
UZ_10	2	no	V	1.5 (musty)	2.2
UZ_19	2	no	L	4.8 (fusty-muddy sediment)	0
UZ_21	2	no	L	4.3 (fusty-muddy sediment)	0
UZ_22	2	no	L	5.1 (rancid)	0
UZ_24	2	no	EV	0.0	4.2

Table S1c. Sensory results (expressed as mean of the medians of intensities provided by each panel) of samples from the first year (180 oils subdivided in 4 sub-groups). *n.c*, not classified; Mpd, main perceived defect. Third sub-group.

Sample code	Selection n.	Formative reassessment	Quality grades	Mpd (intensity and type)	Fruity (intensity)
EU_2	3	no	V	1.4 (winey-vinegary)	2.7
EU_5	3	no	V	1.6 (fusty-muddy sediment)	1.8
EU_12	3	***yes***	V	1.5 (rancid)	1.2
EU_13	3	no	EV	0.0	4.7
EU_16	3	no	V	2.2 (fusty-muddy sediment)	2.6
EU_27	3	no	V	1.8 (brine)	2.3
EU_29	3	***yes***	V	***Misalignment on the mpd***	/
EU_30	3	no	EV	0.0	3.7
EU_32	3	***yes***	V	2.1 (fusty-muddy sediment)	2.0
EU_35	3	no	V	1.7 (fusty-muddy sediment)	2.5
IP_2	3	no	EV	0.0	4.9
IP_4	3	no	EV	0.0	4.4
IP_7	3	no	EV	0.0	3.9
IP_10	3	no	EV	0.0	4.6
IP_17	3	no	V	1.7 (fusty-muddy sediment)	1.3
IP_18	3	no	V	1.2 (fusty-muddy sediment)	1.3
IP_23	3	no	V	2.1 (fusty-muddy sediment)	2.0
IP_26	3	no	L	6.6 (fusty-muddy sediment)	0
IP_29	3	no	L	4.2 (rancid)	0.3
IP_31	3	no	V	2.5 (fusty-muddy sediment)	1.5
IT_3	3	no	EV	0.0	4.0
IT_6	3	no	EV	0.0	3.9
UN_4	3	no	EV	0.0	4.6
UN_6	3	***yes***	V	1.2 (rancid)	2.4
UN_8	3	no	EV	0.0	4.5
UN_12	3	no	EV	0.0	4.9
UN_13	3	no	V	1.2 (winey-vinegary)	2.2
UN_18	3	no	V	1.9 (fusty-muddy sediment); 1.9 (musty)	1.6
UN_23	3	no	V	2.4 (winey-vinegary)	1.9
UN_31	3	no	V	1.9 (fusty-muddy sediment)	1.9
UN_34	3	no	EV	0.0	4.3
UN_39	3	no	V	2.5 (fusty-muddy sediment)	2.2
UP_4	3	no	EV	0.0	4.5
UP_8	3	no	V	1.8 (rancid)	1.7
UP_9	3	no	L	4.1 (rancid)	0
UP_13	3	no	V	1.5 (fusty-muddy sediment)	1.7
UP_17	3	no	EV	0.0	4.4
UP_18	3	no	EV	0.0	3.6
UP_21	3	no	EV	0.0	3.2
UP_24	3	no	EV	0.0	5.3
UP_25	3	no	V	2.0 (rancid)	2.4
UP_29	3	***yes***	V	2.6 (fusty-muddy sediment)	2.3
UZ_7	3	no	L	3.7 (rancid)	0.3
UZ_8	3	no	EV	0.0	2.8
UZ_9	3	no	EV	0.0	3.4

UZ_12	3	no	L	5.4 (rancid)	0
UZ_14	3	no	L	4.6 (fusty-muddy sediment)	0
UZ_15	3	***yes***	V	1.9 (rancid)	1.4
UZ_16	3	***yes***	V	6.5 (fusty-muddy sediment); 1.6 (rancid)	1.7
UZ_18	3	no	EV	0.0	3.9

Table S1d. Sensory results (expressed as mean of the medians of intensities provided by each panel) of samples from the first year (180 oils subdivided in 4 sub-groups). *n.c*, not classified; Mpd, main perceived defect. Fourth sub-group.

Sample code	Selection n.	Formative reassessment	Quality grades	Mpd (intensity and type)	Fruity (intensity)
EU_4	4	*yes*	V	1.6 (rancid)	2.0
EU_6	4	no	L	5.6 (fusty-muddy sediment)	0.2
EU_9	4	no	L	4.6 (musty)	0.0
EU_10	4	*yes*	L	3.8 (fusty-muddy sediment)	0.3
EU_11	4	no	EV	0.0	4.5
EU_15	4	no	V	2.1 (rancid)	2.0
EU_18	4	no	L	5.2 (fusty-muddy sediment)	0.3
EU_19	4	*yes*	V	1.8 (fusty-muddy sediment)	2.5
EU_20	4	no	EV	0.0	4.4
EU_25	4	no	L	6.3 (fusty-muddy sediment)	0.3
IP_3	4	no	EV	0.0	4.4
IP_6	4	no	EV	0.0	3.5
IP_11	4	no	EV	0.0	4.5
IP_19	4	*yes*	L	3.8 (fusty-muddy sediment)	0.5
IP_21	4	no	V	2.4 (rancid)	2.4
IP_28	4	no	L	4.8 (rancid)	0
UN_5	4	no	EV	0.0	4.1
UN_10	4	*yes*	*n.c*	***Misalignment on the category***	/
UN_11	4	no	EV	0.0	4.0
UN_14	4	*yes*	L	3.9 (fusty-muddy sediment)	0.5
UN_16	4	no	L	4.8 (rancid)	0.4
UN_21	4	no	V	2.6 (frostbitten)	2.7
UN_25	4	*yes*	V	1.6 (rancid)	1.7
UN_26	4	no	V	2.0 (musty)	1.6
UN_29	4	no	EV	0.0	4.1
UN_30	4	no	EV	0.0	4.8
UN_35	4	*yes*	L	4.1 (rancid)	0.9
UN_36	4	no	V	2.2 (fusty-muddy sediment)	1.8
UP_14	4	*yes*	*n.c*	***Misalignment on the category***	/
UP_16	4	no	V	2.5 (rancid)	2.2
UP_26	4	no	L	4.1 (rancid)	0.3
UZ_25	4	*yes*	L	5.0 (rancid)	0
UZ_26	4	no	V	2.2 (fusty-muddy sediment)	2.1
UZ_27	4	no	L	3.9 (rancid)	0.6

Table S2a. Sensory results (expressed as mean of the medians of intensities provided by each panel) of samples from the second year (154 oils subdivided in 4 sub-groups). *n.c*, not classified; Mpd, main perceived defect. First sub-group.

Sample code	Selection n.	Formative reassessment	Quality grades	Mpd (intensity and type)	Fruity (intensity)
EU_59	1	no	V	1.7 (fusty-muddy sediment)	2.1
EU_63	1	no	EV	0.0	4.4
EU_64	1	no	V	1.5 (fusty-muddy sediment)	2.8
EU_65	1	no	V	1.3 (rancid)	2.7
EU_72	1	*yes*	V	2.2 (rancid)	2.0
EU_76	1	no	EV	0.0	4.4
IP_33	1	no	EV	0.0	5.3
IP_41	1	no	EV	0.0	4.9
IP_42	1	no	V	3.3 (musty)	0.3
IP_45	1	no	V	2.3 (rancid)	2.7
IP_55	1	no	L	5.1 (fusty-muddy sediment)	0.2
IP_57	1	*yes*	V	2.6 (rancid)	1.3
IT_15	1	no	V	1.8 (rancid)	1.8
IT_20	1	no	V	2.4 (fusty-muddy sediment)	2.6
IT_27	1	no	L	5.2 (fusty-muddy sediment)	0.3
IT_34	1	no	EV	0.0	3.1
IT_35	1	no	V	1.7 (winey-vinegary)	2.9
IT_38	1	no	V	1.1 (rancid)	1.7
IT_41	1	no	EV	0.0	2.9
UN_42	1	no	EV	0.0	3.0
UN_43	1	no	V	2.6 (musty)	0.7
UN_44	1	no	V	1.9 (fusty-muddy sediment)	1.8
UN_55	1	no	V	1.4 (rancid)	1.9
UN_56	1	no	EV	0.0	5.1
UN_57	1	no	V	1.7 (fusty-muddy sediment)	3.1
UN_60	1	*yes*	V	2.8 (winey-vinegary)	0.6
UZ_28	1	no	EV	0.0	2.6
UZ_33	1	*yes*	V	2.3 (musty)	1.0
UZ_36	1	no	EV	0.0	4.2
UZ_46	1	no	V	1.8 (winey-vinegary)	1.6
UZ_47	1	no	L	5.5 (fusty-muddy sediment)	0.3
UZ_52	1	no	EV	0.0	3.2
ZRS_1	1	no	***Anomalous due to the presence of lemon flavour***		
ZRS_5	1	no	EV	0.0	5.7
ZRS_16	1	no	EV	0.0	3.2

ZRS_20	1	***yes***	L	4.6 (musty)	0.4
ZRS_22	1	no	L	4.4 (musty)	0.3
ZRS_23	1	***yes***	EV	0.0	1.8

Table S2b Sensory results (expressed as mean of the medians of intensities provided by each panel) of samples from the second year (154 oils subdivided in 4 sub-groups). *n.c*, not classified; Mpd, main perceived defect. Second sub-group.

Sample code	Selection n.	Formative reassessment	Quality grades	Mpd (intensity and type)	Fruity (intensity)
EU_55	2	***yes***	V	2.1 (rancid)	1.4
EU_56	2	no	EV	0.0	2.9
EU_60	2	***yes***	V	1.6 (rancid)	1.4
EU_68	2	no	EV	0.0	4.8
EU_69	2	no	EV	0.0	3.7
EU_70	2	no	V	2.0 (rancid)	1.9
EU_74	2	no	EV	0.0	3.5
EU_77	2	no	EV	0.0	3.0
IP_34	2	no	EV	0.0	4.9
IP_38	2	no	EV	0.0	3.8
IP_46	2	no	L	5.4 (fusty-muddy sediment)	0.0
IP_51	2	no	EV	0.0	3.0
IP_52	2	no	L	4.5 (rancid)	0.6
IP_56	2	no	L	4.9 (fusty-muddy sediment)	0.0
IT_17	2	no	EV	0.0	3.7
IT_19	2	no	EV	0.0	3.4
IT_22	2	no	V	2.1 (rancid)	1.7
IT_29	2	no	V	2.0 (rancid)	2.7
IT_32	2	no	EV	0.0	2.3
UN_46	2	no	EV	0.0	4.0
UN_54	2	no	V	1.5 (fusty-muddy sediment)	2.3
UN_59	2	no	V	2.3 (brine)	2.0
UN_61	2	no	L	5.0 (musty)	0.4
UN_67	2	no	EV	0.0	3.6
UN_68	2	no	EV	0.0	5.1
UZ_34	2	**no**	EV	0.0	2.4
UZ_35	2	*yes*	L	5.4 (musty)	0.0
UZ_38	2	no	L	5.7 (musty)	0.0
UZ_39	2	no	EV	0.0	3.8
UZ_40	2	***yes***	L	4.5 (fusty-muddy sediment); 4.5 (musty)	0.0
UZ_42	2	no	L	5.6 (rancid)	0.3
UZ_44	2	no	V	1.8 (rancid)	2.0
ZRS_4	2	no	EV	0.0	2.9
ZRS_6	2	***yes***	EV	0.0	2.6
ZRS_13	2	***yes***	EV	0.0	3.0
ZRS_14	2	no	EV	0.0	3.5
ZRS_17	2	no	V	2.5 (rancid)	1.7
ZRS_21	2	***yes***	L	5.0 (rancid)	0.0
ZRS_24	2	no	EV	0.0	4.8

Table S2c. Sensory results (expressed as mean of the medians of intensities provided by each panel) of samples from the second year (154 oils subdivided in 4 sub-groups). *n.c*, not classified; Mpd, main perceived defect. Third sub-group.

Sample code	Selection n.	Formative reassessment	Quality grades	Mpd (intensity and type)	Fruity (intensity)
EU_57	3	no	V	1.9 (fusty-muddy sediment)	2.0
EU_61	3	no	EV	0.0	3.7
EU_66	3	no	EV	0.0	1.9
EU_67	3	no	EV	0.0	2.5
IP_35	3	no	EV	0.0	4.4
IP_36	3	no	EV	0.0	4.5
IP_39	3	no	EV	0.0	4.1
IP_43	3	**yes**	EV	0.0	3.5
IP_47	3	no	L	4.7 (fusty-muddy sediment)	0.0
IP_54	3	no	L	5.6 (rancid)	0.0
IT_16	3	no	L	5.7 (fusty-muddy sediment)	0.0
IT_18	3	no	EV	0.0	2.6
IT_24	3	no	EV	0.0	4.1
IT_28	3	no	L	6.6 (fusty-muddy sediment)	0.0
IT_31	3	no	EV	0.0	2.3
IT_36	3	no	EV	0.0	3.4
IT_40	3	no	V	2.0 (rancid)	1.8
IT_42	3	no	V	1.3 (fusty-muddy sediment)	2.4
UN_45	3	no	EV	0.0	3.7
UN_47	3	no	EV	0.0	4.4
UN_49	3	**yes**	V	2.2 (musty)	1.1
UN_62	3	no	L	4.9 (musty)	0.0
UN_63	3	no	L	6.2 (musty)	0.0
UN_66	3	no	EV	0.0	3.7
UN_69	3	no	EV	0.0	3.9
UN_104	3	no	V	2.3 (rancid)	1.5
UZ_29	3	no	L	3.9 (fusty-muddy sediment)	1.1
UZ_30	3	no	L	6.4 (musty)	0.0
UZ_31	3	*yes*	L	4.2 (musty)	0.7
UZ_37	3	*yes*	L	4.4 (rancid)	0.0
UZ_45	3	no	L	5.6 (musty)	0.0
UZ_50	3	no	L	6.8 (fusty-muddy sediment)	0.0
ZRS_2	3	no	EV	0.0	5.1
ZRS_3	3	no	EV	0.0	3.5
ZRS_7	3	no	V	1.8 (rancid)	2.4
ZRS_8	3	no	V	1.7 (fusty-muddy sediment)	2.1
ZRS_9	3	no	EV	0.0	2.7
ZRS_25	3	no	EV	0.0	5.1

Table S2d. Sensory results (expressed as mean of the medians of intensities provided by each panel) of samples from the second year (154 oils subdivided in 4 sub-groups). *n.c,* not classified; Mpd, main perceived defect. Fourth sub-group.

Sample code	Selection n.	Formative reassessment	Quality grades	Mpd (intensity and type)	Fruity (intensity)
EU_71	4	no	EV	0.0	2.6
EU_73	4	no	EV	0.0	5.1
EU_75	4	no	L	5.9 (musty)	0.0
IP_37	4	no	EV	0.0	4.7
IP_40	4	no	EV	0.0	3.0
IP_44	4	no	V	2.5 (fusty-muddy sediment)	2.2
IP_50	4	no	V	1.7 (rancid)	2.1
IP_53	4	no	L	4.8 (fusty-muddy sediment)	0.0
IT_21	4	no	V	2.6 (fusty-muddy sediment)	2.2
IT_23	4	no	EV	0.0	2.6
IT_25	4	no	V	1.7 (winey-vinegary)	3.2
IT_26	4	no	L	4.1 (rancid)	0.0
IT_30	4	no	V	2.1 (winey-vinegary)	2.7
IT_33	4	no	EV	0.0	2.7
IT_37	4	no	EV	0.0	3.3
IT_39	4	no	V	2.4 (musty)	1.8
UN_48	4	no	EV	0.0	3.0
UN_50	4	no	EV	0.0	4.3
UN_51	4	no	V	1.5 (rancid)	2.4
UN_52	4	no	EV	0.0	4.3
UN_53	4	no	V	2.5 (rancid)	2.1
UN_58	4	no	EV	0.0	1.9
UN_64	4	no	L	6.0 (musty)	0.0
UN_65	4	no	EV	0.0	4.9
UN_103	4	no	EV	0.0	2.5
UZ_32	4	no	V	1.1 (rancid)	1.8
UZ_41	4	no	L	5.7 (musty)	0.0
UZ_43	4	no	V	2.0 (rancid)	2.5
UZ_48	4	no	V	1.8 (rancid)	1.6
UZ_49	4	no	V	2.0 (rancid)	1.8
UZ_51	4	no	L	4.4 (rancid)	2.0
ZRS_10	4	no	V	2.0 (fusty-muddy sediment)	2.4
ZRS_11	4	no	V	2.7 (fusty-muddy sediment)	1.8
ZRS_12	4	no	V	2.1 (fusty-muddy sediment)	2.2
ZRS_15	4	no	EV	0.0	4.2
ZRS_18	4	no	EV	0.0	4.3
ZRS_19	4	no	V	1.9 (rancid)	1.8
